# Analyzing the Loss and the Recovery of Consciousness: Functional Connectivity Patterns and Changes in Heart Rate Variability During Propofol-Induced Anesthesia

**DOI:** 10.3389/fnsys.2021.652080

**Published:** 2021-04-06

**Authors:** Davide Sattin, Dunja Duran, Sergio Visintini, Elena Schiaffi, Ferruccio Panzica, Carla Carozzi, Davide Rossi Sebastiano, Elisa Visani, Eleonora Tobaldini, Angelica Carandina, Valeria Citterio, Francesca Giulia Magnani, Martina Cacciatore, Eleonora Orena, Nicola Montano, Dario Caldiroli, Silvana Franceschetti, Mario Picozzi, Leonardi Matilde

**Affiliations:** ^1^Neurology, Public Health, Disability Unit, Fondazione IRCCS Istituto Neurologico Carlo Besta, Milan, Italy; ^2^Clinical and Experimental Medicine and Medical Humanities-PhD Program, Insubria University, Varese, Italy; ^3^Clinical and Experimental Epileptology Division, Fondazione IRCCS Istituto Neurologico Carlo Besta, Milan, Italy; ^4^Department of Neurosurgery, Fondazione IRCCS Istituto Neurologico Carlo Besta, Milan, Italy; ^5^Neurophysiology Unit, Fondazione IRCCS Istituto Neurologico Carlo Besta, Milan, Italy; ^6^Clinical Engineering Unit, Fondazione IRCCS Istituto Neurologico Carlo Besta, Milan, Italy; ^7^Department of Anaesthesia, Fondazione IRCCS Istituto Neurologico Carlo Besta, Milan, Italy; ^8^Department of Internal Medicine, Fondazione IRCCS Ca’ Granda, Ospedale Maggiore Policlinico, Milan, Italy; ^9^Department of Clinical Sciences and Community Health, University of Milan, Milan, Italy; ^10^Center for Clinical Ethics, Biotechnology and Life Sciences Department, Insubria University, Varese, Italy

**Keywords:** consciousness, anesthesia, connectivity, heart rate variability, propofol

## Abstract

The analysis of the central and the autonomic nervous systems (CNS, ANS) activities during general anesthesia (GA) provides fundamental information for the study of neural processes that support alterations of the consciousness level. In the present pilot study, we analyzed EEG signals and the heart rate (HR) variability (HRV) in a sample of 11 patients undergoing spinal surgery to investigate their CNS and ANS activities during GA obtained with propofol administration. Data were analyzed during different stages of GA: baseline, the first period of anesthetic induction, the period before the loss of consciousness, the first period after propofol discontinuation, and the period before the recovery of consciousness (ROC). In EEG spectral analysis, we found a decrease in posterior alpha and beta power in all cortical areas observed, except the occipital ones, and an increase in delta power, mainly during the induction phase. In EEG connectivity analysis, we found a significant increase of local efficiency index in alpha and delta bands between baseline and loss of consciousness as well as between baseline and ROC in delta band only and a significant reduction of the characteristic path length in alpha band between the baseline and ROC. Moreover, connectivity results showed that in the alpha band there was mainly a progressive increase in the number and in the strength of incoming connections in the frontal region, while in the beta band the parietal region showed mainly a significant increase in the number and in the strength of outcoming connections values. The HRV analysis showed that the induction of anesthesia with propofol was associated with a progressive decrease in complexity and a consequent increase in the regularity indexes and that the anesthetic procedure determined bradycardia which was accompanied by an increase in cardiac sympathetic modulation and a decrease in cardiac parasympathetic modulation during the induction. Overall, the results of this pilot study showed as propofol-induced anesthesia caused modifications on EEG signal, leading to a “rebalance” between long and short-range cortical connections, and had a direct effect on the cardiac system. Our data suggest interesting perspectives for the interactions between the central and autonomic nervous systems for the modulation of the consciousness level.

## Introduction

General anesthesia (GA) is a pharmacologically induced reversible state in which the alterations of the Central and the Autonomic Nervous Systems (CNS, ANS) functioning cause behavioral unresponsiveness and decreased arousal and awareness. The changes induced in consciousness by anesthesia are often used as a theoretical model to study consciousness itself and deepen some issues related to it (e.g., Disorders of Consciousness; Pinsker, 2012; Sanders et al., 2012; Bonhomme et al., 2019). Anesthesiologists perform their work to obtain safe and rapid Loss of Consciousness (LOC) and to realize a proper Recovery of Consciousness (ROC) after surgery to prevent both too deep sedations as well as accidental awareness during GA. However, what exactly happens in both the CNS and ANS during the induction of anesthesia and the recovery is still poorly understood. In the next two paragraphs, we rough out some evidence that emerged in studies using Propofol administration during GA, a sedative-hypnotic agent which involves a positive modulation of the inhibitory function of the neurotransmitter gamma-aminobutyric acid (GABA) through GABA-A receptors (Sebel and Lowdon, 1989), that defined the empirical background of the present article.

### EEG Relative Power, Global and Local Network Patterns During Anesthesia

Regarding the study of the changes in the brain networks, Electroencephalography (EEG) is one of the most used techniques to explore the activity of CNS due to its high temporal resolution and its feasible use in the GA environment. Recent articles focused on exploring the changes in the spectral analysis or in the global or local connectivity during GA showing very interesting data. Spectral analysis, and in particular the analysis of the Relative Power (RP) distribution in various frequency bands, is the most EEG variable studied in the GA environment. It is well-known that arousal changes due to anesthesia are related to significant EEG modifications, namely a decrease in posterior alpha and an increase in fronto-central beta power during the induction phase and an increase in frontal power predominance in alpha, theta, and delta frequencies in deep sedation. These dynamics invert during the ROC and all these effects seemed to be common for different sedative and hypnotic agents (Gugino et al., 2001). Again, Purdon et al. (2013) suggested that the low-frequency phase moderates alpha amplitude to be maximal at low-frequency peaks during deep sedation, whereas maximal at low-frequency nadirs during the transitional phases of loss and recovery of consciousness (Purdon et al., 2013).

Recent studies on global and local network patterns in the brain during GA suggested that the parietal-frontal network, one of the most long-range brain network studied in the scientific literature about consciousness (Lee et al., 2013a,b; Ryu et al., 2017), seemed to reverse the phase relationship between the two regions during propofol administration. A decrease in spectral Granger causality (a probabilistic measure investigating causality between two variables in a time series), for example, from the frontal to parietal areas was observed in the low-frequency bands conversely associated with an increase from the parietal to frontal areas of the high-frequency bands (Kim et al., 2017). In line with this result, propofol administration seemed to cause a disruption of hub-structures in the parietal region, an area that seems to play a pivotal role in information integration and transmission in the brain (Lee et al., 2013a). Lee et al. (2010) stated that changes of feedback and feedforward functional connections in the frontoparietal network are associated with changes in states of human consciousness. Moreover, a recent study on functional networks highlighted the relationship between individual alpha rhythms and unresponsiveness during sedation further reporting as the phase-amplitude coupling between slow and alpha oscillations correlated with propofol concentrations in blood (Chennu et al., 2016).

### Autonomic Nervous System and Heart Rate Variability Changes During Anesthesia

On the other side, the study of ANS signals during general anesthesia is quite difficult, due to the complex systems that regulate sympathetic and parasympathetic balance as well as the limitations regarding the ANS activity measurement due to the extreme fluctuations of the system (Acharya et al., 2006). However, some techniques based on Heart Rate (HR), acquired with electrocardiography (ECG) and the tone of vascular smooth muscle showed good reliability in monitoring the ANS activity even in overstimulating environments such as surgery rooms. One of the commonly used techniques monitoring the ANS activity is the Heart Rate Variability (HRV) which is focused on the HR fluctuation over time (Task Force of the European Society of Cardiology and the North American Society of Pacing and Electrophysiology, 1996).

The application of HRV for the study of the role of ANS for consciousness evaluation is quite recent (Riganello et al., 2019). Some studies showed an increase in High-Frequency component power indicating an increase in vagal control of HR before the loss of consciousness in the GA environment: propofol seemed to reduce the total power as well as Low-Frequency power with an increase in the proportional part of HF power (Storella et al., 1999; Tarvainen et al., 2012). However, the results on the role of ANS on the modifications of consciousness level during GA are still lacking.

### Open Questions and Aims of the Present Study

As pointed out, CNS and ANS could provide important information on both the loss and the recovery of consciousness, using the analysis of both brain networks as well as the balance between sympathetic and vagal systems at different stages of sedation, in the same patients. Indeed, the study of how neural systems generate consciousness implies a profound analysis of what happens in that systems during situations in which consciousness is altered by different agents.

However, despite the results reported above, there are many open questions and limitations regarding CNS and ANS analysis during GA. For example, in most studies, only the baseline and period after LOC are compared using EEG data without considering the changes within the induction or the recovery periods, so giving a partial analysis of the activities of the cortical circuits (Kreuzer et al., 2014; Schneider et al., 2014). Moreover, apart from the frontal and parietal areas, the other cortical areas are less explored, although the role of global and local brain areas connections as well as the characteristics of these connections are highlighted as really important by some recent theories on consciousness (Tononi and Edelman, 1998; McFadden, 2007; Prakash et al., 2008; Yu and Blumenfeld, 2009). Moreover, other theories (Bosse et al., 2008; Damasio, 2012) underline the importance of the ANS as the pivotal systems fundamental for consciousness generation connecting information from the internal changes of the vegetative system to the cortical elaboration of stimuli and *vice versa*. HRV is a real chance to analyze how ANS information changed during LOC and ROC and its analysis during GA, although its analysis is often limited to pain perception (Logier et al., 2006; Sawaguchi et al., 2016; Garret-Bernardin et al., 2017) and not as an opportunity to study the balance between sympathetic and parasympathetic activity during the administration of anesthetic agents considering the changes within the induction or the recovery periods as for EEG analysis.

Therefore, the present pilot study aims to analyze CNS and ANS features through EEG and HRV analysis, in a sample of patients during propofol-induced unconsciousness for spinal surgical treatments. In detail, we investigated EEG spectral and connectivity properties in different bands and brain regions to explore changes during different phases of the induction and recovery processes. In parallel, we performed a symbolic and complexity analysis of the HRV signals to detect non-reciprocal changes of sympathetic and parasympathetic modulation and to evaluate cardiac autonomic complexity. All the changes in EEG and HRV parameters during different phases of the induction and recovery processes were evaluated and results will be discussed in light of recent theories on consciousness.

## Materials and Methods

### Participants

We enrolled 19 adult patients undergoing spinal surgery (for herniation or stenosis) in the neurosurgical unit. Exclusion criteria included age less than 18 years old, CNS disease, cardiovascular disease, or any disease that is known to affect the ANS, or patients taking drugs affecting the ANS, CNS, or cardiovascular system. Exclusion criteria also included a history of alcohol, drug abuse, or major psychiatric diseases. Data on eight patients were excluded from the analysis due to clinical problems during anesthesia that caused the changing of the anesthetic protocol (*n* = 5) or low quality of EEG and HRV collected data during the induction or recovery phases (*n* = 3).

### Study Design and Procedure

**Figure 1:**
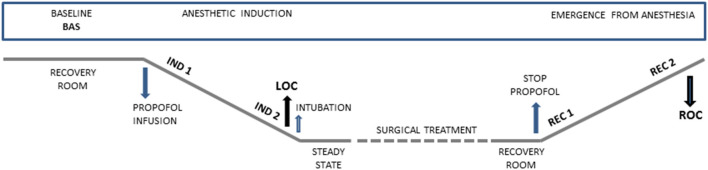
Graphical representation showing time points of data collection during anesthetic induction and emergence from general anesthesia. Bas: Baseline, Ind1: the first period of anesthetic induction after propofol infusion, Ind2: the period before the loss of consciousness (LOC), Rec1: the first period after that the propofol infusion was stopped, Rec2: the period before the recovery of consciousness (ROC).

All patients were enrolled in this prospective observational study during the pre-hospitalization visit (around 15 days before the surgery). During the preparation for the neurosurgical treatment, electrodes for EEG and ECG were applied. The data collection was made in five different conditions: at baseline (Bas), during the induction of anesthesia from the start of propofol infusion (Ind1) and immediately before the LOC (Ind2), and during the recovery phase immediately after the interruption of the propofol infusion (Rec1) and until the complete ROC (Rec2). LOC was defined according to the Observer Assessment of Alertness/Sedation Scale (OAA/S; Chernik et al., 1990). The timeline of the acquisition is reported in Figure 1.

The study was approved by the local Ethics Committee and written informed consent was obtained from all the participants.

### General Anesthesia Protocol and Monitoring

The following protocol is routinely applied by the anesthesiologist’s staff to all patients who undergo surgery in our center. The study was conducted in a fully-equipped recovery room and in the operating theatre with three-lead ECG, pulse oximetry, capnography, and non-invasive blood pressure monitoring. An intravenous cannula was inserted in the forearm for fluids and intravenous drug administration. Intravenous GA was administered through a Bispectral Index (BIS)-guided anesthesia protocol where hypnotic/analgesic dosages were continuously adjusted according to the EEG depressive effect of drugs and to clinical levels of sedation. A BIS-xp electrode was placed on the subject’s forehead, according to the manufacturer’s recommendations, and was connected to the BIS Vista monitor (BIS Vista, Medtronic, MN, USA).

Each BIS index range, between 100 and 0, is clinically correlated to a sedation level as depicted by OAA/S. The interval 100–80 correlates to fully awake/minimally sedated, i.e., a patient able to open spontaneously the eyes, the 80–60 correlates to light/moderate sedation, i.e., a patient that opens his eyes after the command to open. The 60–40 correlates to LOC, i.e., the general anesthesia ideal plane where the patient does not open the eyes after mild prodding (level 1 of the OAA/S scale) and is associated with the appearance of a showed delta rhythm plus spindle-like waves with the absence of fast waves, features of general anesthesia (Bennett et al., 2009). Below 40 BIS number corresponds to over sedation.

Anesthesia was induced by mean of TCI delivery system (Injectomat^®^ TIVA Agila, Fresenius Kabi, France) settled at Effect-site Concentration (Ce), i.e., at the Cerebral Concentration desired. According to the pharmacokinetic model for propofol (Schnider et al., 1999) and Remifentanil (Minto et al., 1997), the software pump calculates the infusion rate of drugs to achieve and maintain the chosen Ce at any time, avoiding overshoot or under dosage during dose adjustments and drug accumulation.

Starting from a propofol Ce of 2 μg/ml, the Ce was increased by 1 μg/ml after 1 min of equilibration at the reached Ce, until the appearance of LOC. The Remifentanil infusion started after the LOC to prevent pain for intubation, while at ROC the Ce Remi was recorded (range Ce 1–1.5 μg/ml). Then curare (cisatracurium) was administered, and the patient was intubated (0.1–0.2 mg/kg of Cisatracurium is the usual dosage for intubation with the routine use of Glidescope at our Institution). In the present study, the cisatracurium was administered only once after LOC for intubation purposes. Since the Recovery time of Curarization is around 50–60 min for Cisatracurium, any effect could be largely negligible at ROC. All patients were extubated without reversal drugs of curarization (mean anesthesia duration exceeded 60 min). The surgery phase of anesthesia was managed for maintaining stable standard clinical parameters (BIS 40-60, blood pressure, heart rate, and absence of body movement). At the end of the surgical procedure, the patient was then transferred to the recovery room for the awakening, and propofol infusion was stopped, while Remifentanil was reduced at Ce 1 ng/ml for additional analgesia and tolerance to the orotracheal tube. The propofol and Remifentanil decay were continuously monitored on the pumps. After reaching the ROC, the patients were extubated, and Remifentanil stopped.

### Signal Acquisition

#### EEG

EEG was recorded using 19 Ag/AgCl surface electrodes (impedance <5 kΩ) placed following the 10-20 International System and acquired at a sampling rate of 256 Hz (Micromed SpA, Mogliano Veneto, Treviso, Italy). In four patients, the signal acquired on Fz had a very low signal-to-noise ratio thus only 18 common channels were included in the analysis. Data were average re-referenced and band-pass filtered in 0.5–48 Hz band. The analysis was performed on 1-min artifact-free EEG representative epochs acquired in the five conditions (Bas, Ind1, Ind2, Rec1, and Rec2; Figure 1).

#### ECG

To evaluate cardiac autonomic control during the different stages of anesthesia, the ECG signals were recorded (SystemPLUS, Micromed S.p.A., Treviso, IT, USA) in the different conditions following the scheme reported in Figure 1. ECG recordings were processed through a specific software (Heartscope II; AMPS-LLC, New York, NY, USA). The R-R intervals between adjacent R peaks were identified to provide the tachogram. Ectopic beats were deleted from the resulting R-R series and replaced by a virtual beat placed by interpolating adjacent R peaks as recommended (Task Force of the European Society of Cardiology and the North American Society of Pacing and Electrophysiology, 1996). Finally, segments of 250 ± 50 consecutive beats were selected for each period. We applied two different methods for the analysis of HRV on the selected segments, symbolic analysis and complexity analysis as described in the next sections.

### EEG Analysis

#### Spectral Power Analysis

The EEG epochs were filtered (1–40 Hz, 12 db/octave) and, for each condition, divided into 30 non-overlapping 2-s segments and analyzed using the fast Fourier transform (FFT). Relative power (RP) was calculated in the delta (1–4 Hz), theta (4–8 Hz), alpha (8–13 Hz), and beta bands (13–30 Hz), and averaged within each EEG channel.

#### Connectivity Analysis

The connectivity pattern was studied using Partial Directed Coherence (PDC), a frequency-domain measure derived from the multivariate autoregressive (MVAR) modeling of multichannel EEG signals (Baccalá and Sameshima, 2001). The characteristic of the PDC is to capture the direction of the influence and to be less affected by the volume conduction problem than other methods.

For each condition, EEG data were epoched in non-overlapped short segments of 3 s which were de-trended and normalized by subtracting the mean value and dividing the result by the variance. The length of the epoch was chosen to balance the stationarity of the data and the model fit (better parameter estimation for longer segments). All of the EEG channels were simultaneously used as inputs for the MVAR model. The optimal order was chosen after calculating for each epoch the Bayesian information criterion. All segments showed the optimal model order ranging from 25 to 30, so order 26 was chosen and applied to all the epochs. Once the MVAR coefficients had been adequately estimated, the PDC spectra were estimated. The statistical significance of non-zero PDC values, at each frequency, was assessed by a bootstrap approach using phase randomization. Only significant connections were considered and averaged over segments. PDC values were calculated in the same bands considered for the spectral power analysis. To evaluate local connectivity patterns, in addition to the mean global PDC value, regional PDC values were calculated by averaging values into five regions of interest (ROIs): frontal (F3, F4, Fp1, and Fp2), central (C3, C4, and Cz), parietal (P3, P4, Pz), occipital (O1 and O2) and temporal (T4, T6, F8, T5, T3, F7).

Graph theory was applied to describe connectivity results by calculating indices describing the topological properties of the networks. To compare connectivity indices, a fixed number of connections was set for each band as the minimum level of connections to have a connected network in all subjects.

Global efficiency, local efficiency, the characteristic path length, and the clustering coefficient measured the level of integration and segregation of the whole network. Moreover, the number of connections (degree) and the strength of connections as well as the directional values in/out-degree, in/out-strength (number and strength of incoming and outgoing connections), and in/out-degree fraction were calculated by averaging values of the electrodes within ROIs.

The signals were pre-processed and analyzed using a custom-written toolbox in MATLAB (MathWorks Inc., Natick, MA, USA) using open-source toolboxes (Fieldtrip, BCT, eMVAR). A detailed description of the indexes used was reported in the Supplementary Materials.

### ECG Analysis

#### Symbolic Analysis

Cardiac autonomic control was evaluated through a non-linear approach to detect non-reciprocal changes of sympathetic and parasympathetic modulation (Porta et al., 2001, 2007a,b; Guzzetti et al., 2005). After the transformation of the tachogram into a sequence of symbols (i.e., words), four patterns were identified and quantified as to their rate of occurrence considering sequences of three consecutive symbols: 0 V%, when a sequence of three symbols shows no variations; 1 V%, pattern with only one variation; 2 LV%, when the symbols show two like variations (ascending or descending slope); 2 UV%, the pattern with two unliked variations. Experimental and clinical studies involving pharmacological blockade and autonomic tests reveal that 0 V% index is a marker of cardiac sympathetic modulation while 2 LV% and 2 UV% indexes are markers related to cardiac vagal modulation (Porta et al., 2001, 2007a,b; Guzzetti et al., 2005).

#### Complexity Analysis

The heart rate variability is the result of the activity of several mechanisms acting over similar but not coincident frequencies (e.g., vagal and sympathetic modulation, baroreflex and chemoreflex regulation, respiratory sinus arrhythmia, hormonal influences, local factors). All these mechanisms contribute to the complexity of the signal and non-linear tools, such as entropy-derived measures, provide information on the complexity of the autonomic cardiac control in both physiological and pathophysiological conditions (Porta et al., 2001). In this study, we applied Corrected Conditional Entropy (CCE) to evaluate cardiac autonomic complexity. CCE is derived from Conditional Entropy that assesses the amount of information carried by the current RR sample. CCE is bounded between zero when the new sample is fully predictable, and Shannon entropy, when the new sample is unpredictable. By dividing CCE by the Shannon entropy, it is possible to derive an index of regularity (Ro) which is bounded between 1, maximum regularity and lowest complexity, to 0, lowest regularity, and maximum complexity (Porta et al., 2001).

### Statistical Analysis

Statistical analyses were performed using SPSS (version 17, SPSS Inc. Chicago, IL, USA) and SigmaStat software (2016 Systat Software, Inc., Chicago, IL, USA). Repeated measures analysis of variance (RM-ANOVA) was applied to assess the effects of the anesthesia conditions on RP, PDC, connectivity values, in each band separately, and on cardiovascular autonomic measures. The sphericity assumption was evaluated using Mauchly’s test and the Greenhouse-Geisser correction was applied when appropriate. The Shapiro–Wilk test was used to assess the normality of data distribution. When a non-normal distribution was found, differences were examined by the Friedman Test for repeated measures analysis of variance on ranks. *Post-hoc* analyses were performed using paired *t*-test (Bonferroni corrected). Statistical significance was always set at *p* < 0.05 for all the analyses.

## Results

The characteristics of the patients are shown in Table 1. No intraoperative awareness or recall was reported by any patient in the study.

**Table 1:** Demographic and clinical data.

Patient id	Gender	Age (years)	Weight (Kg)	Height (cm)	Diagnosis
1	F	66	57	160	Spinal disc herniation L4, L5
2	F	53	57	160	Spinal disc herniation L4, L5
3	M	49	80	175	Spinal disc herniation L5, S1
4	M	67	70	168	Spinal disc herniation L3, L4
5	F	56	59	160	Spinal disc herniation L5, S1
6	M	62	71	169	Spinal stenosis L3, L4
7	M	79	95	180	Spinal stenosis L2, L3
8	F	74	93	175	Spinal stenosis L3, L4
9	F	45	49	160	Spinal stenosis L5, S1
10	M	30	78	170	Spinal disc herniation L4, L5
11	M	76	91	169	Spinal stenosis L3-L5

### EEG Relative Power

**Figure 2:**
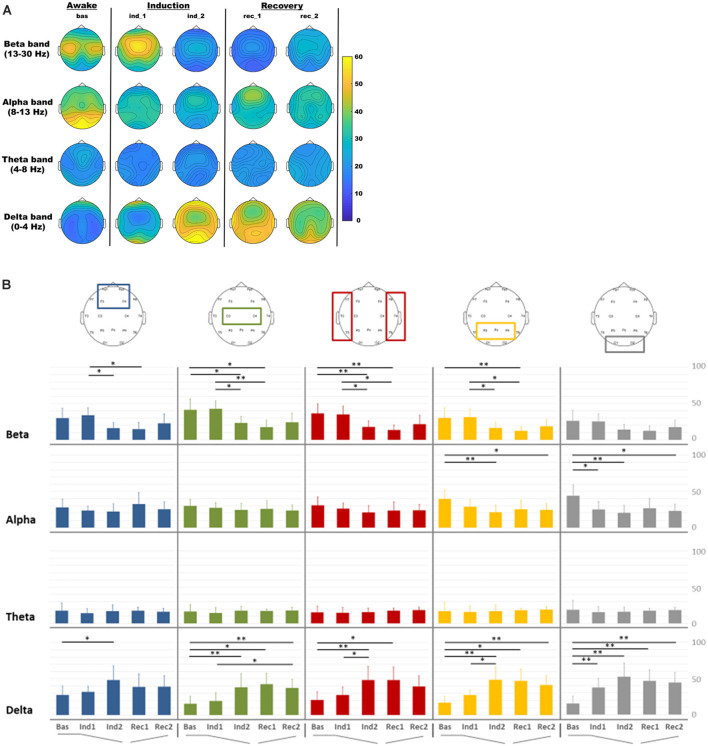
Electroencephalography (EEG) relative power distribution for delta, theta, alpha, and beta bands in each condition. **(A)** EEG relative power maps. **(B)** EEG relative spectral power comparisons among regions of interest (ROIs) for each band analyzed in each condition: baseline (Bas), beginning phase of administration of propofol (Ind1), loss of consciousness (Ind2), propofol infusion stopped (Rec1) and pre-recovery of consciousness (Rec2) period for frontal (blue), central (green), temporal (red), parietal (orange) and occipital (gray) ROIs. *p < 0.05; **p < 0.01.

In Figure 2A was reported the EEG RP distribution for delta, theta, alpha, and beta bands in each condition. During the induction and recovery period, a decrease in occipital alpha RP was observed, whereas an increase in delta RP was noted in all regions. Central beta RP increased during the beginning period of the induction and then decreased in the other intervals. The statistical comparison of RP in different periods among frequency bands and ROIs was reported in Figure 2B.

RM-ANOVA for ROIs and intervals indicated significant changes in delta (*p* < 0.001), alpha (*p* < 0.001), and beta (*p* = 0.009) bands. *Post-hoc* investigation of delta band in all regions showed that relative power in delta band increased in all ROIs during the induction intervals, with significant differences between Bas and Ind2 (pre-LOC) in all regions. The occipital region only showed a significant difference between baseline and light sedation (Ind1) in the delta (*p* < 0.001), whereas differences between the light (Ind1) and deep sedation (Ind2) were seen in temporal (*p* = 0.027) and parietal (*p* = 0.037) ROIs. During the recovery step, it is worth noting that this increase in delta activity did not recover to the initial values observed during the baseline step as high relative power persisted in the pre-ROC (Rec2) interval compared to pre-anesthesia state for central (*p* = 0.004), parietal (*p* = 0.005), and occipital (*p* = 0.001) ROIs.

Alpha and beta band revealed, on the other side, a significant reduction in relative power during the induction intervals. In the alpha band this pattern was observed mainly in posterior ROIs during the induction phase (Bas vs. Ind2; parietal, *p* = 0.008 and occipital, *p* = 0.004) and persisted with a significant difference in the same areas between the Bas and Rec2 period (*p* < 0.05 for both areas). In the beta band, this decrease during the induction phase was evident especially in Ind1 and Ind2 for almost all ROIs, except the occipital region.

### Graph Global Properties

**Figure 3:**
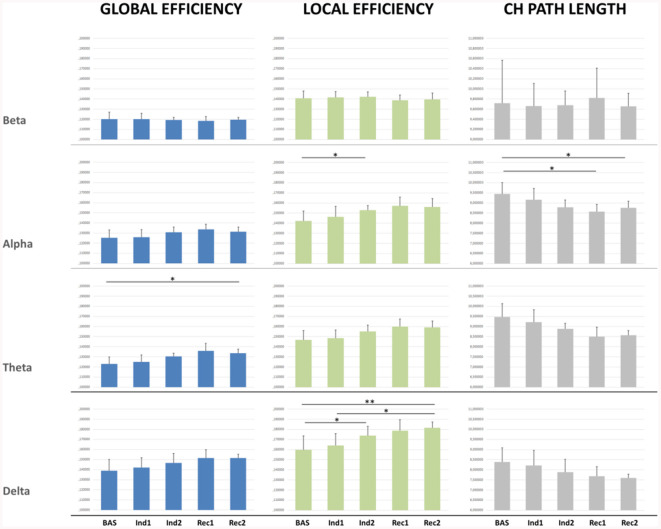
Connectivity indexes measured for the whole brain network for each EEG band in each condition.*p < 0.05; **p < 0.01.

Significant results were found mainly in the lower frequency bands (Figure 3). Global efficiency changed significantly in the delta (*p* = 0.007), theta (*p* < 0.001), and alpha band (*p* = 0.013). *Post-hoc* pairwise comparison showed an increasing trend during the sedation compared to the baseline, not recovering to the initial baseline value in the awakening (*p* = 0.03) for the theta band.

Similarly, significant changes were observed also in Local Efficiency in delta (*p* < 0.001) theta (*p* < 0.001), and alpha band (*p* < 0.01). *Post-hoc* pairwise comparisons showed a significant increase from Bas to Ind2 interval in delta (*p* = 0.04) and alpha band (*p* = 0.03) indicating a decrease in the distance among neighboring nodes and so an increased efficacy in the information transfer within regions during the induction phase. During the recovery, the LE did not return to the baseline value in the delta band (*p* = 0.005) showing an increasing trend.

Characteristic path length confirmed an increase of network connections in lower bands, showing decreasing trend values from Bas to Ind2 in the alpha band, maintaining the lower values during all recovery from anesthesia, and not returning to the initial values in the pre-ROC interval (Bas vs. Rec2, *p* = 0.03). Cluster Coefficient did not show any significant statistical differences comparing different stages and bands.

### Regional Graph Properties

RM-ANOVA showed significant results for most of the considered network indexes (in/out/total-degree and in/out/total-strength) in theta, alpha, and beta bands. In the beta band, post-hoc ANOVA for the effect of the interval demonstrated that, in particular, parietal ROI was involved in the change, covering all the period of sedation and recovery (*p* < 0.001); indeed, the pairwise comparison showed a significant increase of total-degree (Bas-Rec2: *p* = 0.03). This increment was confirmed observing also in the parietal out-degree index (*p* < 0.001) during induction, with *post-hoc* significant changes found between Bas-Ind2 (*p* = 0.02), Bas-Rec1 (*p* = 0.04), and Ind1-Rec1 (*p* = 0.02). In/out-Degree fraction (*p* < 0.001) highlighted that, during emergence from anesthesia, the network properties of the awake state are not recovered (Bas- Rec1, *p* < 0.001 and Bas-Rec2, *p* = 0.004). On the other side, in the frontal region, a decreasing number of connections between the light sedation and the awakening (Ind1-Rec2, *p* = 0.041) was observed.

**Figure 4:**
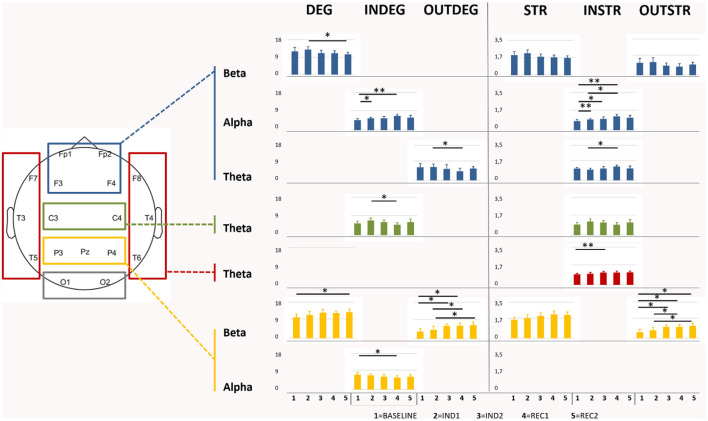
Graphical representation of the significant differences of degree (DEG), Strength (STR), and the directional in/out-degree (INDEG/OUTDEG) and strength (INSTR/OUTSTR) values in each ROIs for each anesthesia period and EEG bands. Note: frontal area showed an increase in the in indexes in the alpha bands during the induction revealing different statistical differences between some periods whereas the statistically significant comparisons in the out indexes were highlighted in the beta band in the parietal area. *p < 0.05; **p < 0.01.

In the alpha band, frontal and parietal ROIs displayed an opposite pattern, according to the in-degree value. Frontal regions increased the number and strength of the incoming connections during induction, with a highly significant change of in-strength index (Bas-Ind1: *p* < 0.001; Bas-Ind2: *p* = 0.037; Bas-Rec1: *p* < 0.001; Ind1-Rec1: *p* = 0.01), and of in-degree values (Bas-Ind1: *p* = 0.01; Bas-Rec1: *p* = 0.003). On the other side, parietal ROI revealed a decrease of in-degree from Bas to Rec1 interval (*p* = 0.04). Finally, in the theta band, a decrease in the number of outgoing connections (*p* = 0.03) and an increase in the strength of the incoming connections (*p* = 0.02) between Ind1 and Rec1 was observed in frontal ROI. In temporal ROI, in-strength increased (Bas-Ind2: *p* = 0.009), while in central ROI, the number of incoming connections decreased (Ind1-Rec1, *p* = 0.01). All ANOVAs and comparisons that resulted in statistically significant values were reported in Figure 4.

### ECG Analysis

**Figure 5:**
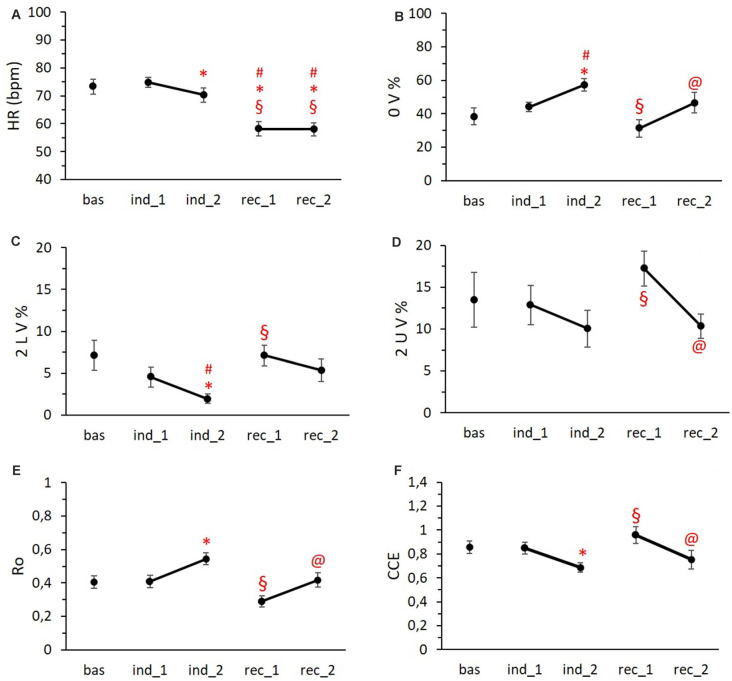
HR trend **(A)**, symbolic metrics trends **(B,C,D)**, and complexity metrics trends **(E,F)** during the different phases of the protocol. Data are reported as means ± standard errors. ^#^vs. Bas, *vs. Ind1 ^§^vs. Ind1, ^@^vs. Rec1. Abbreviations: HR = heart rate; 0 V% = pattern with no variations; 2 LV% = pattern with two like variations; 2 UV% = pattern with two unlike variations; Ro = index of regularity; CCE = corrected conditional entropy.

Regarding the analysis of the ECG data, we observed a progressive decrease in the mean Heart Rate starting from the second induction period with significant bradycardia during the two phases of recovery (Figure 5A; *p* < 0.001). Concerning the temporal course of cardiovascular autonomic control, the symbolic analysis revealed an increase of pattern with no variation (0 V%; Figure 5B), a marker of cardiac sympathetic modulation, in both induction phases reaching the significance in Ind2 (*p* = 0.045), while the first recovery period was characterized by values of 0 V% significantly lower than Ind2 (*p* = 0.002). Conversely, the parasympathetic indices (2 LV% and 2 UV%) presented an opposite trend with a progressive reduction during the induction phases, followed by an increase in the first recovery period and a final return to baseline values (Figures 5C,D). Specifically, the frequency of 2 LV% in Ind2 was significantly lower compared to 2 LV% Bas values (*p* < 0.05) and 2 LV% values recorded in Rec1. Similarly, 2 UV% in Ind2 differed significantly from those obtained in the Rec1 period (*p* < 0.05).

As to complexity measures, the index of regularity (Ro) showed an increase during the last period of induction, dropped below the baseline value in the first recovery period, and, at last, we observed a return to baseline values (Figure 5E). Complementarily, CCE was decreased in the second phase of induction and overshot the baseline values during Rec1 (Figure 5F). We found a significant difference between Ind2 and Rec1 for both Ro and CCE mean values (*p* < 0.001 and *p* = 0.006, respectively).

As to the temporal differences inside each condition, despite a lower mean Heart Rate, Ind2 was characterized by a higher sympathetic and lower parasympathetic modulation (*p* = 0.012 and *p* < 0.001, respectively; Figures 5B,C) compared to Ind1. The same trend was present in the two recovery phases: Rec2 showed higher 0 V% values (Figure 5B, *p* = 0.013) and lower 2 UV% values (Figure 5D, *p* = 0.004) compared to Rec1. Finally, both the second phases of induction and recovery period showed greater regularity (Figure 5E, *p* = 0.007 and *p* < 0.001) and less complexity (Figure 5F, *p* = 0.021 and *p* = 0.026) than the first phases.

## Discussion

In this work, we studied the changes of EEG and HRV features during induction and recovery from general anesthesia as a theoretical model for the understanding of consciousness.

In summary, the main results are:

In the *EEG spectral analysis*: (i) a decrease in posterior alpha and beta power in all areas except for the occipital ones and an increase in delta power mainly during the induction phase; (ii) no statistically significant variation in the theta band across periods and areas.

In *EEG connectivity analysis*: (iii) a significant increase of local efficiency index in alpha and delta bands between baseline and LOC as well as between baseline and ROC in delta band only, whereas the global efficiency index did not reveal any statistical differences except between baseline and ROC in the theta band; (iv) a significant reduction of the characteristic path length in alpha band between the baseline and ROC. Moreover, our data showed that; (v) in the alpha band there was mainly a progressive increase in the number and the strength of incoming connections in the frontal ROI, while in the beta band the parietal ROI showed mainly a significant increase in the number and the strength of outcoming connections values with a persistent trend in the recovery period; and (vi) the theta band pattern resulted statistically different in different areas, with a statistically significant increase of the strength of incoming connections during the induction phase in the temporal area.

The *HRV analysis* showed that: (vii) the induction of anesthesia with propofol was associated with a progressive decrease in complexity (CCE) and a consequent increase in the regularity index Ro of the RR series; (viii) the anesthetic procedure determined bradycardia which was accompanied by an increase in cardiac sympathetic modulation (0 V%) and a decrease in cardiac parasympathetic modulation (2 LV%) during the induction; (ix) all indexes returned to basal values during the recovery period except for heart rate in contrast to the most analyzed EEG indexes. In the next paragraphs, all these results are thoroughly discussed.

### EEG Spectral Analysis

It is well-known that arousal changes following anesthesia with propofol are related to significant EEG modifications, although a direct compound among studies is difficult due to the different EEG epochs analyzed and the external variables influencing electrical activities present in a complex environment like recovery/surgical rooms.

The decrease in posterior alpha power is found also by other studies confirming that our result is in line with other researches (Gugino et al., 2001; Yeom et al., 2017). In contrast, beta power tends to increase initially, after administration of propofol, and then decrease significantly during the induction phase showing an effect that is debated in the literature (Gugino et al., 2001; John et al., 2001), and that seemed to be correlated to the direct action of propofol. Regarding the increase in slow (i.e., theta and delta) and very low (under 1 Hz) frequencies associated with the appearance of frontal alpha frequencies in deep sedation (CIT), our data confirmed these results although the very low frequencies were not analyzed in our study. These dynamics reverse during the recovery, although with an asymmetric timeframe as already described in the literature (Gugino et al., 2001), confirming that changes in the EEG bands could follow a different trend in the recovery period with respect to the induction one probably due to pharmacokinetic of anesthetic drugs. Indeed, during ROC Opioids and Remifentanil have a variable effect on the EEG because they predominantly affect no cortical structures. Larger doses can produce EEG slowing (until delta waves). A study shows a significant linear correlation between increasing remifentanil concentration (0.5–10 ng/ml) and decreasing of BIS (Koitabashi et al., 2002) and another study found that the effect of remifentanil on the spectrum and quantitative parameters of the electroencephalogram is significant and strongly dependent on the level of anesthesia: (1) decrease of activity in high frequencies (>14 Hz) during light anesthesia; and (2) increase of activity in the extended alpha band (7–14 Hz) and decrease of activity in the delta band (0.5–4 Hz) during deep anesthesia (Bennett et al., 2009). Both studies cannot evaluate the effect of remifentanil alone, since conducted during general anesthesia with propofol and interaction (Propofol /Remi) cannot be excluded. We think that minimal Remifentanil dosages (as at ROC in our study) should not affect the EEG signals but this is a limitation of our study that we reported in the limit section.

John et al. (2001) demonstrated a significant change in hemispheric interactions during the loss of consciousness and sedation, with a “close coupling” of the anterior regions, paralleled by a clear anteriorization of power spectra. The same authors described the “uncoupling” of other regions (anterior and posterior regions on each hemisphere, as well as homologous regions between the two hemispheres). Finally, Soplata et al. (2017) described the transition between consciousness and unconsciousness, highlighting the spectral properties of the EEG, including the alpha background activity (8–12 Hz), the slow-wave oscillation (0.1–1.5 Hz), and the dose-dependent phase-amplitude between them. In this model, the thalamus could have a pivotal role in propofol-induced anesthesia, generating a novel hyperpolarizing very-low-frequency rhythm probably due to a GABAergic effect on the cortex. Our results replicate these previous works, even with slight differences: We did not compare the dynamics of the two hemispheres because we grouped both left and right frontal, central, temporal, parietal, and occipital channels in the same ROIs. Moreover, our result is in line with previous works (Gugino et al., 2001; Yeom et al., 2017) as functional changes in brain dynamics are dissimilar during forward and reverse transition from one conscious state to the other. This complex phenomenon is known as hysteresis, it has been described for both the spectral and the connectivity measures (Kim et al., 2018), and it could be accounted to the pharmacokinetic properties of anesthetic drugs (Kuizenga et al., 2018), although some aspects remain poorly understood (Bonhomme et al., 2019). Also, patients who underwent anesthesia showed a huge heterogeneity (greater than voluntary subjects) while recovering consciousness after drug discontinuation (Chander et al., 2014), potentially increasing the hysteretic effect.

### EEG Connectivity, Global and Local Indexes

Boussen et al. (2018) studied the effect of the re-emergence of consciousness from anesthesia directly on the cortex (through electrocorticography), demonstrating that the emergence states correspond to a simple signal transition in the frequency space, which is possible to be reproduced by a time rescaling of cortical activity during the unconscious state. The authors enhanced the role of the intracortical interactions in the ROC, limiting the thalamus in controlling or modulating the inhibition. Moreover, Lee and colleagues demonstrated that different anesthetics, including propofol, have the main effect to disrupt frontal-parietal networks (Lee et al., 2010, 2013a,b); at the same time, brain networks showed a significant reduction in the number of network connections during anesthesia, although maintaining a scale-free organization across consciousness, anesthesia, and recovery (Lee et al., 2010). On the contrary, a recent study (Vlisides et al., 2019) stated that the alpha band frontal-parietal connectivity was not able to distinguish levels of consciousness or anesthetic states in surgical patients as a single measure, implicitly reducing the importance of the parieto-frontal connections as a “marker of consciousness.”

Since a single measure of functional connectivity is probably not a reliable correlate of the effect induced by the anesthetic (Vlisides et al., 2019), in our work we considered network measures and connectivity indices for each ROI in the classical EEG band. Global efficiency, an index related to the efficiency of communication among all nodes in a network, showed, in the theta band, a significant difference between baseline and pre-ROC. Theta connectivity showed a complex pattern characterized by a decrease of the outgoing connections and an increase of the strength of the incoming connections from induction to initial recovery in the frontal region, with a concurrent decrease in incoming connections in the central region, while an increase of the strength of the incoming connections was observed in temporal ROI. This result appears noticeable because the theta band is the only one among the EEG bands that did not show significant differences in the spectral analysis, revealing that the connectivity and spectral analyses can provide complementary information. In the delta band, local efficiency showed a significant increase between baseline and induction, between baseline and beginning of the recovery, and between the first period of sedation and initial recovery. These results are in line with previous literature reporting that the appearance of theta and the decrease of delta activity characterize the transition from deep to light sedation (Sheeba et al., 2008). Furthermore, our data showed that in the alpha band there was an increase in the number and the strength of incoming connections in the frontal ROI from baseline to induction and initial recovery with a concurrent decrease of the number of incoming connections in the parietal ROI. In the same band, local efficiency showed a significant increase between baseline and deeper sedation state and a significant reduction of the characteristic path length in the alpha band between the baseline and ROC.

Taken together, our results highlighted that: (a) brain networks exhibit a complex, non-linear (i.e., the recovery process is a different and slower period than the loss of consciousness process) pattern of modifications during anesthesia; (b) these modifications affect several frequency bands of the EEG signal; (c) leading to a “rebalance” between long and short-range cortical connections, and finally, that (d) different ROIs, namely frontal and parietal ones, show opposite behavior. This endorses the hypothesis of disrupted “long-range” networking from frontal to temporal and parietal areas (Supp et al., 2011) with the resulting prevalence of short-range connections during the loss of consciousness and recovery during the emergence period, albeit in a different range of time, with a longer ROC period.

### HRV Analysis

The analysis of the ANS showed that: (i) the induction of anesthesia with propofol was associated with a decrease in complexity, with a consequent increase in the regularity index, and in cardiac parasympathetic modulation; (ii) the anesthetic procedure determined bradycardia which was accompanied by an increase in cardiac sympathetic modulation during the induction; and (iii) me more regular and coherent. Indeed, Hutt the return to basal values was rather complete of all indexes during the recovery period except for heart rate.

General anesthesia determines a global depression of CNS functions, as well as the cardiovascular autonomic functions. As it has been previously reported in the literature, the nature and characteristics of the cardiovascular autonomic control alterations directly depend on the type and dose of anesthetic drugs (Ebert, 2005; Riznyk et al., 2005; Mäenpää et al., 2011). Despite different effects of the anesthetics on the two branches of ANS, to the best of our knowledge, the majority of studies confirms a general decrease in HRV during the induction phases due to the progressive reduction in the complexity of cardiovascular control systems (Scheffer et al., 1993; Kanaya et al., 2003; Tarvainen et al., 2012; Guzzetti et al., 2015a,b). These observations are in line with our data suggesting a reduction in the complexity of the physiological mechanisms regulating heart rate, as demonstrated by the reduction of CCE and the increase of the regularity index Ro.

The propofol administration resulted in a slowing of the HR during the induction phase, accompanied by an increase in cardiac sympathetic and reduction in cardiac vagal modulation. General anesthetic doses of propofol are associated with bradycardia, which is associated with low catecholamine plasma levels (Mustola et al., 1995) and with hypotension, which is the consequence of a direct effect of propofol on venous smooth muscle tone and presumably venous return (Cullen et al., 1987; Muzi et al., 1992; Koch et al., 2008). Moreover, propofol seems to act at the cardiac level with a negative inotropic effect by depressing myocardial contractility (Riou et al., 1992; Cook and Housmans, 1994; Bilotta et al., 2001). Therefore, as per previous studies (Scheffer et al., 1993; Guzzetti et al., 2015a,b), our observations seem to confirm a direct effect of the propofol on the heart, resulting in a cardiac sympathetic activation and a reduction in vagal modulation in response to hemodynamic alterations, i.e., the effects on peripheral vascular control as a calcium antagonist.

As for the changes found in the transition from the second phase of induction to the first phase of recovery, it cannot be excluded that they are the result of the surgical intervention (Hirata et al., 2012). Further insights and the use of continuous ECG recordings are required to better understand the observed differences between the induction and awakening phases.

Finally, it is important to underline that there was a return of all the HRV parameters to the basal values, except for the HR. Despite a general recovery of autonomic functions, our data indicate that propofol-induced bradycardia may persist after discontinuing the infusion. Other studies have observed lasting effects of propofol on cardiovascular control with a resolution of the microcirculatory alterations 3 h after discontinuation of infusion (Kamijo et al., 1992; Koch et al., 2008) and that Remifentanil effect on HRV is possible since Remifentanil stimulates the parasympathetic nervous system but the effects of opioids on HRV remain poorly characterized. So future studies will take into consideration these effects also, considering that in the absence of nociception, HRV total power is reduced during GA with an increase of the proportional part of HF and that with Remifentanil, HF decreases after nociceptive stimulation (Jeanne et al., 2009).

### Overview and Future Perspectives

Considering both EEG and HRV results in a wider framework, our data seem to support different theories on general anesthesia as well as on consciousness itself.

The so-called theory of the “consciousness switch” (Alkire et al., 2000) proposes that the mechanism supporting the loss of consciousness is a hyperpolarization block of thalamocortical neurons, due to a GABA neurotransmission enhancement and glutamate and cholinergic neurotransmission inhibition in the thalamocortical loops. A recent interesting perspective affirmed that anesthetic action results in an effective decrease in broadband neural activity with the neural structures involved become more regular and coherent. Indeed, Hutt et al. (2018) observed that weak fluctuations in the brain circuit caused a nonlinear oscillatory instability, yielding local intra-area synchrony in the α frequency band. This intra-area synchrony disrupts synchrony between cortex and thalamus, with a relay role of the lateral geniculate nucleus, and contributes to the loss of consciousness. Moreover, it has been shown that a disruption of this system can drive thalamic nuclei into an attractor state of low-frequency bursting and further entrainment of thalamocortical circuits, with a characteristic downward shift of dominant α-peaks in the EEG power spectra, together with increased power over the lower frequencies (van Wijngaarden et al., 2016). This is in line with the modification in EEG we obtained, i.e., the decrease in posterior alpha and, after an initial increase in fronto-central beta power due to propofol and then a decrease of it, followed by an increase in slow frequencies typically observed in deep sedation and in thalamocortical disconnection.

The theory affirmed also that the precuneus and the adjacent areas within the posteromedial parietal cortex may play a central role as neural correlates of consciousness and self-reflection processes (Cavanna, 2007). Our results did not analyze the thalamocortical disconnection directly, but our findings revealed that the outgoing connectivity of the parietal region increased in the beta band in terms of both degree and strength, giving the impression that these nodes increased their information outflow processing during the induction phase of the anesthesia. Another system that should be taken into account for the consciousness and arousal modulation is the thalamic reticular activating system comprises the ascending reticular activating system (ARAS) and its thalamic targets, mainly intralaminar nuclei. The mesencephalic ARAS nuclei cover an array of structures, and experiments in mice have shown that the central lateral nucleus and ventrobasal nucleus co-operate to modulate the thalamocortical loops involved in temporal binding synchronizing cortical neuronal discharges as reported above (Llinás et al., 2002).

Considering the role of ANS, different results found in studies analyzing the changes in the HRV values during attentional tasks seemed to reveal that an increase in cardiac sympathetic modulation is counterbalanced by an increased vagal tone (Blase and van Waning, 2019). Our results are different as we highlighted a reduction in cardiac vagal modulation. However, the debate on the role of ANS during a performance that required focal or sustained attention is still controversial: Lower HRV is associated with worse attentional performance (Duschek et al., 2009; Williams et al., 2016), although these results were not confirmed by Frewen et al. (2013). The Neurovisceral Integration Hypothesis (Thayer and Lane, 2000, 2009), another interesting theory that tries to understand the mechanisms that allow consciousness generation, suggested that the brain areas involved in self-regulation and executive functions are also involved in cardiac autonomic activity through the vagus nerve (Thayer and Sternberg, 2006; Thayer et al., 2010), although the real mechanisms behind this association are far to be clear. An example of this activity is the so-called baroreceptor reflex, which coordinates cardiac output with blood pressure. A primary function of the cardiovascular system is to maintain optimal arterial blood pressure and to provide adequate blood flow to the brain, and other organs. The baroreceptor reflex involves lower brainstem nuclei that detect organ-specific visceral activity *via* afferent sympathetic and parasympathetic nerve pathways, using this information in homeostatic, reflex-like regulation of autonomic tone. The Neurovisceral Integration Hypothesis supports the idea that new types of information are hierarchically integrated between the vagal system, brainstem, and some cortical areas, suggesting that each level is more flexibly recruited to modify vagal tone than the level below (Thayer and Lane, 2000, 2009). The inverse relationship between HRV and blood pressure variability is an example of an important physiological regulation strategy known as heterostasis (Davis, 1958), where variability in HRV is associated with stability in the blood pressure system. The hierarchical, prolonged and coordinated information transmission of blood pressure system with information derived from brainstem nuclei (e.g., nucleus ambiguous, the dorsal motor nucleus of the vagus, nucleus of the solitary tract) and other brain structures (e.g., amygdala and the basal forebrain) seems to be involved in the regulations of EEG bands (Sanchez-Alavez et al., 2014), as well as seems to be fundamental for downward predictions and for minimizing error in motor tasks, for example (Smith et al., 2017). It is premature to infer specific causal relationships among these systems and our results but a deep analysis of the interactions among these systems could be useful in the future.

Another interesting result is that the processes involved in the loss of consciousness seem to be different from the ones related to the recovery phase. This creates a new perspective according to the two phases of general anesthesia that should be studied separately: the mechanisms linked to the “switch on” of consciousness/attention could be not the same as the “switch off” process (Uhrig et al., 2014). Indeed, in our results, the changes in outgoing connectivity values of beta bands in parietal ROI were not restored during the recovery phase confirming how, as reported also by other authors (Blain-Moraes et al., 2017), the CNS (mainly) and ANS (for heart rate) mechanisms could be different during the loss and recovery of consciousness. If this result will be confirmed, the monitoring systems of the anesthesia during the two phases could be different in the near future to increase the sensitivity, with (e.g.,) a focus on the posterior electrical activity of the brain during the induction phase (when possible) and a deep monitor of the HRV, instead, during the recovery phase.

Finally, another interesting theory called Global Neuronal Workspace (GNW; Dehaene et al., 1998; Dehaene and Changeux, 2011), explaining the brain mechanisms behind disorders of consciousness, affirmed that cortico-cortical long-range axons in prefronto-parietotemporo-cingulate cortex constitute the brain network through which the primary sensory information became conscious. The anesthetic drug seems to cause a widespread cortical activity disruption in the neurons of these areas with long-distance connectivity, while the delay for thalamic activity disruption is related to the indirect consequences of cortical feedback on the thalamus (Velly et al., 2007). Our results seem to confirm this evidence considering that we found an increase in the local efficiency value during the administration of propofol in alpha e delta bands with no statistically significant differences in global efficiency. Nevertheless, an explanation of the mechanisms through which these activities were associated with the results from ANS data along with the progressive bradycardia due to anesthesia is still lacking and it needs to be analyzed in future studies focusing on Central and Autonomic nervous systems correlates of consciousness (Riganello et al., 2019; Sattin et al., 2020).

### Limits

This pilot study has some limitations that should be considered. First, the number of patients analyzed was small and we cannot draw any general conclusions. Furthermore, several variables should be taken into account to limit their confounding role; for instance, the neurosurgical and reanimation settings required careful work to limit the confounding variables influencing data acquisition, and single-subject variability in response to anesthetic agents should be considered as well. However, the aim of this pilot study was only to explore the differences among epochs both in the CNS and in the ANS during the propofol-induced anesthesia through some measures that gave us interesting perspectives that should be tested in future studies with adequate sample size. Another limitation of our study was that the total amount of Remifentanil administered during GA was not recorded and this information is lacking in the discussion about the recovery period, although we monitored the Ce range in according to our GA protocol. However, we think that minimal Remifentanil dosages (as at ROC in our study) should not affect the EEG signals.

Regarding limitations in EEG analyses, PDC and DTF, were used as they did not take into account instantaneous connectivity. The choice of the reference and volume conduction influence is still debatable, while some claim that DTF and PDC can indeed disentangle the true connectivity (Kaminski and Blinowska, 2014), even if spurious connections are present (Kaminski and Blinowska, 2017), others (Brunner et al., 2016) proved otherwise. The volume conduction error could affect the analysis on sensors and connectivity should have been done on source data. Unfortunately, with only 19 channels, source reconstruction could not be performed. In future studies, an HD-EEG or MEG should be used instead, besides source reconstruction and volume correction methods.

Furthermore, a limitation was related to the fact that we analyzed the EEG and ECG signals in a parallel way. Future studies are needed to combine all these signals.

Among the other limitations that could be highlighted in this pilot study, we want finally to underline that we only considered propofol anesthetic in our research and, hence, every general conclusion about brain mechanisms or neural processes should be cautiously discussed. Indeed, some studies reported as other drugs act on brain networks in different ways concerning propofol (Vakkuri et al., 2004; Nicolaou and Georgiou, 2014) producing the same loss of consciousness fundamental for a surgery. In this sense, future studies are needed to verify the association between CNS and ANS changes during the loss and recovery of consciousness in a wider sample.

### Conclusion

In conclusion, the results of this pilot study showed as propofol-induced anesthesia administration caused modifications affecting several frequency bands of the EEG signal, leading to a “rebalance” between long and short-range cortical connections with an increase in local efficiency in alpha e delta bands during the induction phase. Moreover, different ROIs, namely frontal and parietal ones, showed opposite effects with an increase in the incoming values in the alpha bands during the induction of anesthesia and a statistically significant comparison in the outgoing values in the beta bands, respectively. Finally, the induction of anesthesia with propofol determined a progressive decrease in the complexity of the HRV and bradycardia which was accompanied by an increase in cardiac sympathetic modulation and a decrease in cardiac parasympathetic modulation during the induction phase followed by a return to basal except for the heart rate index, suggesting interesting perspectives for the interactions between central and autonomic nervous system during anesthesia.

## Data Availability Statement

The datasets presented in this study can be found in online repositories. The names of the repository/repositories and accession number(s) can be found below: https://zenodo.org/communities/besta/?page=1&size=20.

## Ethics Statement

The studies involving human participants were reviewed and approved by Local Ethics Committee Fondazione IRCCS Istituto Neurologico Carlo Besta. The patients/participants provided their written informed consent to participate in this study.

## Author Contributions

DS and DD: conceptualization, data interpretation, original draft writing, and critical revision of the manuscript draft. DD, ES, FP, DRS, EV, SF, ET, AC, VC, and NM: data collection, data analyses and interpretation, and critical revision of the manuscript draft. SV, CC, EO, and DC: data collection and interpretation, critical revision of the manuscript draft. FGM and MC: data interpretation and critical revision of the manuscript draft. MP and LM: conceptualization and critical revision of the manuscript draft.

## Conflict of Interest

The authors declare that the research was conducted in the absence of any commercial or financial relationships that could be construed as a potential conflict of interest.
