# Supplementary material for: Analyzing the Loss and the Recovery of Consciousness: Functional Connectivity Patterns and Changes in Heart Rate Variability During Propofol-Induced Anesthesia
Source: Front Syst Neurosci. 2021 Apr 6;15:652080. doi: 10.3389/fnsys.2021.652080 (PMC8055941; doi:10.3389/fnsys.2021.652080)
Supplement: Supplementary file 1 [file Table_1.DOCX]

**Supplementary material**

We employed graph theory approach to describe connectivity results, defining the network as an abstract representation of a set of vertices (or nodes) linked by means of edges (or connections). Using this definition, each element 𝑎𝑖𝑗 of the adjacency matrix A is different from 0 if there is an effective link between nodes 𝑖 and 𝑗 and equal to 0 otherwise. Several indices can be computed for the characterization of the properties of the networks [The Brain Connectivity Toolbox (brain-connectivity-toolbox.net)]. In this study, we used a weighted version of the indices, which account also for the strength and direction of the connectivity links returned by PDC. [DOI: 10.1016/j.neuroimage.2009.10.003][doi:10.1162/NECO_a_00914]

Connectivity measures used to describe the global properties of the graph were **Weighted Characteristic path length** (mean shortest path length), **Weighted** **Global Efficiency** and **Weighted Mean local efficiency**.

In order to define those measures of integration, we need to map distance from weights (we used the simplest version $l_{ij}=1/{w_{ij}})$; we can define then the shortest weighted path from *i* to *j*, $g_{i\to j}^{w}$ . The shortest weighted path length is then calculated as

$$d_{ij}^{w}=\sum_{a_{uv\varepsilon g_{i\to j}^{w}}} l_{uv}$$

**Weighted Characteristic Path Length**. The characteristic path length is the average shortest path length in the network. Its weighted and directed version can be defined as follows:

$$L^{w}=\frac{1}{n}\sum_{i\varepsilon N} \frac{\sum_{j\varepsilon N,j\neq i} d_{ij}^{w}}{n-1}$$

**Weighted Global Efficiency**. The global efficiency represents the efficiency of the communication between all the nodes in the network and it is calculated as the average of the inverse of the geodesic length (shortest path between two nodes in the network). In case of weighted and directed network we thus obtain:

$$E^{w}=\frac{1}{n}\sum_{i\varepsilon N} \frac{\sum_{j\varepsilon N,j\neq i} {{(d}_{ij}^{w})}^{-1}}{n-1}$$

**Weighted Local Efficiency.** Local efficiency is the average of the global efficiencies computed on each subgraph belonging to the network and represents the efficiency of the communication between all the nodes around the node 𝑖 in the network. The weighted and directed version is

$$E_{loc}^{w}\left( i \right)=\frac{1}{max{(W)}^{1/3}}\frac{\sum_{j,h} w_{ij}^{1/3} w_{ih}^{1/3} \left[ d_{jh}^{w} \left( N_{i}^{'} \right) \right]^{-1}}{\sum_{j,h} w_{ij}^{1/3} w_{ih}^{1/3}}$$

where $d_{jh}^{w} \left( N_{i}^{'} \right)$is the adapted shortest distance between *j* and *h* . This adapted shortest distance was calculated as the shortest distance in the network *N* i containing all neighbours of *i* excluding node *i* after replacing the weight of edge *(j, h)* to $w_{jh}^{'}=w_{jh}^{1/3}$ .[Wang et al.2016]

In our study we did not found significant regional differences, thus we calculated mean local efficiency for all the nodes N of the network.

Considering the low number of nodes (electrodes), we calculated only Degree and Strength for each node and we successively averaged the values in each of the five regions considered.

**Degree** The Degree of a node is the total number of links connected to that node *i* ,calculated as:

$$k_{i}=\sum_{j\varepsilon N} a_{ij}$$

and in case of directed network , we can distinguish between **In-degree** (number of incoming links) and **Out-degree** (number of outgoing links ) of a node *i*

**Node Strength**. The strength 𝑠 defines the total intensity of a node i, considering the weights for all the incoming connections (**In-strength**, 𝑠in) and outgoing ones (**Out-strength**, 𝑠out):

$s\left( i \right)= sin (i) + sout (i) =\sum_{j\varepsilon N} w_{ji}+\sum_{j\varepsilon N} w_{ij}$

where 𝑤𝑖𝑗 is the weight of the connection from node i to node j.

**Table 1S.** Cardiovascular autonomic control assessed by heart rate variability parameters during anesthesia phases

| Index | Baseline | induction 1 | induction 2 | recovery 1 | recovery 2 | *p* |
| --- | --- | --- | --- | --- | --- | --- |
| HR (bpm) | 73 ± 9 | 75 ± 6 | 70 ± 9 | 58 ± 9 | 58 ± 8 | < 0.001 |
| Symbolic Analysis | | | | | |  |
| - 0V% | 38.4 ± 16.6 | 43.9 ± 9.3 | 57.3 ± 12.1 | 31.3 ± 17.5 | 46.6 ± 20.3 | 0.005 |
| - 2LV% | 7.1 ± 5.9 | 4.5 ± 4.0 | 1.9 ± 1.9 | 7.1 ± 4.1 | 5.4 ± 4.5 | 0.009 |
| - 2UV% | 13.5 ± 10.9 | 12.9 ± 7.7 | 10.0 ± 7.2 | 17.2 ± 6.9 | 10.3 ± 4.8 | 0.034 |
| COMPLEXITY ANALYSIS | | | | | |  |
| CCE | 0.86 ± 0.17 | 0.85 ± 0.17 | 0.69 ± 0.13 | 0.96 ± 0.24 | 0.75 ± 0.26 | 0.008 |
| Ro | 0.41 ± 0.12 | 0.42 ± 0.12 | 0.54 ± 0.12 | 0.29 ± 0.11 | 0.42 ± 0.15 | < 0.001 |

Abbreviations: HR = heart rate; 0V% = pattern with no variations, index of sympathetic modulation; 2LV% = pattern with two like variations, index of parasympathetic modulation; 2UV% = pattern with two unlike variations, index of parasympathetic modulation; Ro = index of regularity; CCE = corrected conditional entropy, index of complexity.
